# Dichlorido[2-(3,5-dimethyl-1*H*-pyrazol-1-yl-κ*N* ^2^)ethanamine-κ*N*]zinc(II)

**DOI:** 10.1107/S1600536811044217

**Published:** 2011-10-29

**Authors:** Ilia A. Guzei, Lara C. Spencer, Tebogo V. Segapelo, James Darkwa

**Affiliations:** a Department of Chemistry, University of Wisconsin-Madison, 1101 University Ave, Madison, WI 53706, USA; b Department of Chemistry, University of Johannesburg, Auckland Park Kingsway Campus, Johannesburg 2006, South Africa

## Abstract

The amine title complex, [ZnCl_2_(C_7_H_13_N_3_)], resulted from imine hydrolysis in a Schiff base compound. The Zn metal atom has a distorted tetra­hedral geometry with the most significant deviation identified in the magnitude of the N—Zn—N angle. This deviation stems from the participation of the Zn and N atoms in a six-membered metallocyclic ring. The latter is in an approximate screw-boat conformation. Two strong N—H⋯Cl hydrogen bonds link the mol­ecules into ribbons propagating along the *b*-axis direction. The ribbons contain two second-order hydrogen-bonded motifs: a chain and a ring. The chain described by the graph set notation *C*_2_^2^(6) is formed by one hydrogen bond going in the forward direction (donor to acceptor) and the other in the backward direction (acceptor to donor). In the ring motif *R*_2_^2^(8), both hydrogen bonds propagate in the forward direction.

## Related literature

For imine hydrolysis in Schiff base compounds, see: Guzei *et al.* (2010 ▶); Czaun *et al.* (2010 ▶); Bu *et al.* (1997 ▶); Koner & Ray (2008 ▶); Sinha *et al.* (2003 ▶). For graph-set analysis, see: Bernstein *et al.* (1995 ▶). Related structures were found from the Cambridge Structural Database (Allen, 2002 ▶). Bond distances and angles were confirmed to be typical by a *Mogul* structural check (Bruno *et al.*, 2002 ▶). For ring analysis, see: Cremer & Pople (1975 ▶). 
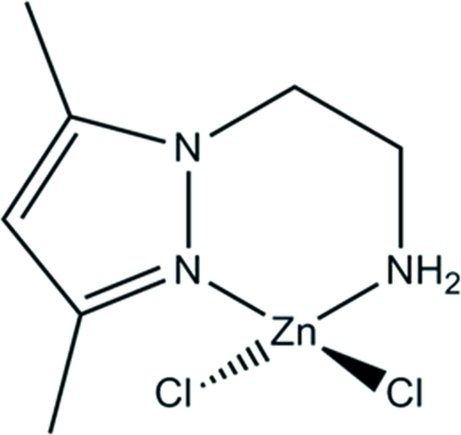


## Experimental

### 

#### Crystal data

[ZnCl_2_(C_7_H_13_N_3_)]
*M* *_r_* = 275.47
Monoclinic, 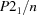

*a* = 9.060 (3) Å
*b* = 8.894 (2) Å
*c* = 14.260 (4) Å
β = 97.95 (3)°
*V* = 1138.1 (6) Å^3^
*Z* = 4
Cu *K*α radiation
μ = 7.00 mm^−1^
*T* = 100 K
0.48 × 0.28 × 0.21 mm

#### Data collection

Bruker SMART APEXII area-detector diffractometer
Absorption correction: multi-scan (*SADABS*; Bruker, 2007 ▶) *T*_min_ = 0.134, *T*_max_ = 0.321
16711 measured reflections
2123 independent reflections
2053 reflections with *I* > 2σ(*I*)
*R*_int_ = 0.019

#### Refinement

*R*[*F* ^2^ > 2σ(*F* ^2^)] = 0.020
*wR*(*F* ^2^) = 0.051
*S* = 1.03
2123 reflections
120 parameters
H-atom parameters constrained
Δρ_max_ = 0.31 e Å^−3^
Δρ_min_ = −0.19 e Å^−3^

### 

Data collection: *APEX2* (Bruker, 2007 ▶); cell refinement: *SAINT* (Bruker, 2007 ▶); data reduction: *SAINT*; program(s) used to solve structure: *SHELXTL* (Sheldrick, 2008 ▶); program(s) used to refine structure: *SHELXTL*, *FCF_filter* (Guzei, 2007 ▶) and *INSerter* (Guzei, 2007 ▶); molecular graphics: *SHELXTL* and *DIAMOND* (Brandenburg, 1999 ▶); software used to prepare material for publication: *SHELXTL*, *publCIF* (Westrip, 2010 ▶) and *modiCIFer* (Guzei, 2007 ▶).

## Supplementary Material

Crystal structure: contains datablock(s) global, I. DOI: 10.1107/S1600536811044217/zq2129sup1.cif

Structure factors: contains datablock(s) I. DOI: 10.1107/S1600536811044217/zq2129Isup2.hkl

Additional supplementary materials:  crystallographic information; 3D view; checkCIF report

## Figures and Tables

**Table:** 

Zn1—N1	2.0214 (13)
Zn1—N3	2.0461 (14)
Zn1—Cl1	2.2266 (6)
Zn1—Cl2	2.2512 (6)

**Table:** 

N1—Zn1—N3	96.88 (6)
N1—Zn1—Cl1	113.62 (4)
N3—Zn1—Cl1	114.24 (4)
N1—Zn1—Cl2	114.15 (4)
N3—Zn1—Cl2	106.77 (4)
Cl1—Zn1—Cl2	110.44 (3)

**Table 2:** Hydrogen-bond geometry (Å, °)

*D*—H⋯*A*	*D*—H	H⋯*A*	*D*⋯*A*	*D*—H⋯*A*
N3—H3*A*⋯Cl2^i^	0.92	2.41	3.3073 (15)	165
N3—H3*B*⋯Cl1^ii^	0.92	2.43	3.2620 (15)	150

Symmetry codes: (i) 
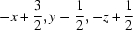
; (ii) 
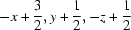
.

**Fig. 1.**
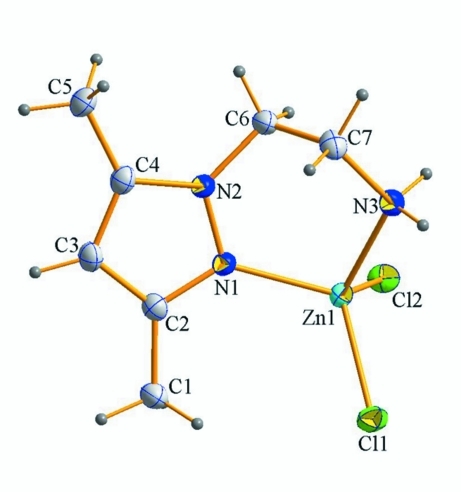
Molecular structure of (I). The thermal ellipsoids are shown at 50% probability level.

## supplementary crystallographic information

### Comment

Imine hydrolysis in Schiff base compounds is quite common. It is usually driven by traces of water and the presence of acidic metal ions, especially in the case of the first row transition metal chlorides such as Co^II^ (Guzei *et al.*, 2010), Ni^II^ (Czaun *et al.*, 2010) and Cu^II^ (Bu *et al.*, 1997; Czaun *et al.*, 2010; Koner & Ray, 2008; Sinha *et al.*, 2003). In a recent attempt to prepare a Zn^II^ complex from the reaction of 2-{[2-(3,5-dimethylpyrazol-1-yl)ethylimino]}-4,6-ditertbutylphenol with zinc(II) chloride, we isolated the title compound (I), a hydrolysis product of the imine to an amine.

The coordination environment of the central metal Zn1 is distorted tetrahedral with angles ranging from 96.88 (6)° to 114.24 (4)°. The dihedral angle between the planes defined by atoms Zn1, N1, N3 and Zn1, Cl1, Cl2 spans 86.77 (4)°. The most significant deviations from the ideal tetrahedral geometry is observed in the N1—Zn1—N3 angle of 96.88 (6)°. This significant deviation is due to its inclusion in a six-membered metallocyclic ring. This ring, Zn1—N1—N2—C6—C7—N3, approaches a screw-boat conformation ^5^S_4_ with the puckering coordinates θ = 74.44 (12)° and φ = 203.47 (13)° (Cremer & Pople, 1975). Data mining of the Cambridge Structural Database (August 2011 update; Allen, 2002) revealed 55 complexes in which a zinc atom is bonded to two chlorine atoms, one nitrogen atom of a pyrazole, and one other nitrogen atom. The complexes may contain metallocyclic rings with five, six, seven, eight, ten, thirteen, or sixteen atoms as well as no metallocyclic ring. The degree of deviation of the N—Zn—N bond angle from the ideal tetrahedral value depends greatly on the number of atoms in the metallocyclic ring. For the 22 complexes in which no ring is formed, the N—Zn—N angle averages of 111 (8)°. The eight complexes with five-membered metallocyclic rings have an average N—Zn—N angle of 79.3 (7)°. For the 18 complexes with six-membered metallocyclic rings the N—Zn—N angle averages 94 (3)°, which compares well to that of compound (I). The N—Zn—N angles in the seven complexes that have greater than six-membered metallocyclic rings average 107 (3)°. All other geometrical parameters of (I) are typical as confirmed by a *Mogul* structural check (Bruno *et al.*, 2002).

Two strong intermolecular hydrogen bonding interactions N3—H3b···Cl1 [-*x* + 3/2, *y* + 1/2, -*z* + 1/2] (a) and N3—H3a···Cl2 [-*x* + 3/2, *y* - 1/2, -*z* + 1/2] (b) link molecules of (I) in chains parallel to the *b* axis. The chains form an  motif (the arrows above the bond designators show the direction of the bond; the forward arrow corresponds to the donor-to-acceptor direction whereas the backward arrow to the acceptor-to-donor direction) described by the second order graph set notation C^2^_2_(6) (Bernstein *et al.* 1995). The hydrogen bonds also form an  ring motif described by the second order graph set notation *R*^2^_2_(8) with both bonds in the forward direction.

### Experimental

A CH_2_Cl_2_ solution (10 ml) of 2-{[2-(3,5-dimethylpyrazol-1-yl)ethylimino]}-4,6-ditertbutylphenol (0.90 g, 2.7 mmol) was added to a CH_2_Cl_2_ suspension (20 ml) of ZnCl_2_ (0.35 g, 2.6 mmol) and stirred at room temperature for 18 h after which a colorless solution was formed. The solution was concentrated in *vacuo* to about 10 ml and hexane added (5 ml) and kept at -4°C to produce crystals of the title compound. Yield: 0.33 g (49%). Anal. Calcd for C_7_H_13_Cl_2_N_3_Zn: C, 32.15; H, 5.01; N, 10.71. Found: C, 32.24; H, 4.89; N, 10.61%.

### Refinement

All H-atoms attached to carbon atoms were placed in idealized locations and refined as riding with appropriate thermal displacement coefficients *U*_iso_(H) = 1.5 times *U*_eq_(bearing atom) for H atoms attached to nitrogen atoms and *U*_iso_(H) = 1.2 times *U*_eq_(bearing atom) for all other H atoms. Default effective *X*—H distances for T = -173.0° C C(*sp* 3)–2H=0.99, C(*sp* 3)–3H=0.98, C(*sp* 2)–H=0.95, N-2H=0.92 Å.

### Crystal data

**Table:**

[ZnCl_2_(C_7_H_13_N_3_)]	*F*(000) = 560
*M**_r_* = 275.47	*D*_x_ = 1.608 Mg m^−^^3^
Monoclinic, *P*2_1_/*n*	Cu *K*α radiation, λ = 1.54178 Å
Hall symbol: -P 2yn	Cell parameters from 9893 reflections
*a* = 9.060 (3) Å	θ = 4.9–69.5°
*b* = 8.894 (2) Å	µ = 7.00 mm^−^^1^
*c* = 14.260 (4) Å	*T* = 100 K
β = 97.95 (3)°	Block, colourless
*V* = 1138.1 (6) Å^3^	0.48 × 0.28 × 0.21 mm
*Z* = 4	

### Data collection

**Table:**

Bruker SMART APEXII area-detector diffractometer	2123 independent reflections
Radiation source: fine-focus sealed tube	2053 reflections with *I* > 2σ(*I*)
graphite	*R*_int_ = 0.019
0.50° ω and 0.5 ° φ scans	θ_max_ = 70.0°, θ_min_ = 5.5°
Absorption correction: multi-scan (*SADABS*; Bruker, 2007)	*h* = −10→10
*T*_min_ = 0.134, *T*_max_ = 0.321	*k* = −10→10
16711 measured reflections	*l* = −17→17

### Refinement

**Table:**

Refinement on *F*^2^	Primary atom site location: structure-invariant direct methods
Least-squares matrix: full	Secondary atom site location: difference Fourier map
*R*[*F*^2^ > 2σ(*F*^2^)] = 0.020	Hydrogen site location: inferred from neighbouring sites
*wR*(*F*^2^) = 0.051	H-atom parameters constrained
*S* = 1.03	*w* = 1/[σ^2^(*F*_o_^2^) + (0.030*P*)^2^ + 0.6489*P*] where *P* = (*F*_o_^2^ + 2*F*_c_^2^)/3
2123 reflections	(Δ/σ)_max_ = 0.001
120 parameters	Δρ_max_ = 0.31 e Å^−^^3^
0 restraints	Δρ_min_ = −0.19 e Å^−^^3^

### Special details

**Table:**

Geometry. All e.s.d.'s (except the e.s.d. in the dihedral angle between two l.s. planes) are estimated using the full covariance matrix. The cell e.s.d.'s are taken into account individually in the estimation of e.s.d.'s in distances, angles and torsion angles; correlations between e.s.d.'s in cell parameters are only used when they are defined by crystal symmetry. An approximate (isotropic) treatment of cell e.s.d.'s is used for estimating e.s.d.'s involving l.s. planes.
Refinement. Refinement of *F*^2^ against ALL reflections. The weighted *R*-factor *wR* and goodness of fit *S* are based on *F*^2^, conventional *R*-factors *R* are based on *F*, with *F* set to zero for negative *F*^2^. The threshold expression of *F*^2^ > σ(*F*^2^) is used only for calculating *R*-factors(gt) *etc*. and is not relevant to the choice of reflections for refinement. *R*-factors based on *F*^2^ are statistically about twice as large as those based on *F*, and *R*- factors based on ALL data will be even larger.

### Fractional atomic coordinates and isotropic or equivalent isotropic displacement parameters (Å^2^)

**Table:**

	*x*	*y*	*z*	*U*_iso_*/*U*_eq_	
Zn1	0.57494 (2)	0.15806 (2)	0.280022 (13)	0.01647 (8)	
Cl1	0.48746 (4)	−0.06529 (4)	0.22820 (3)	0.02530 (10)	
Cl2	0.53128 (4)	0.33130 (4)	0.16443 (3)	0.02379 (10)	
N1	0.50886 (14)	0.22053 (14)	0.40401 (8)	0.0181 (3)	
N2	0.60299 (14)	0.29662 (15)	0.47094 (9)	0.0190 (3)	
N3	0.79922 (14)	0.16142 (14)	0.32554 (9)	0.0197 (3)	
H3A	0.8369	0.0670	0.3173	0.030*	
H3B	0.8433	0.2264	0.2876	0.030*	
C1	0.25483 (18)	0.1137 (2)	0.38927 (12)	0.0251 (3)	
H1A	0.2064	0.1791	0.3389	0.030*	
H1C	0.1835	0.0876	0.4324	0.030*	
H1B	0.2894	0.0217	0.3613	0.030*	
C2	0.38499 (17)	0.19396 (18)	0.44299 (11)	0.0198 (3)	
C3	0.40007 (18)	0.25371 (18)	0.53388 (11)	0.0226 (3)	
H3	0.3282	0.2508	0.5765	0.027*	
C4	0.54005 (18)	0.31816 (18)	0.55017 (11)	0.0213 (3)	
C5	0.61843 (19)	0.3966 (2)	0.63528 (11)	0.0263 (3)	
H5B	0.5498	0.4094	0.6821	0.032*	
H5A	0.6527	0.4953	0.6168	0.032*	
H5C	0.7042	0.3364	0.6628	0.032*	
C6	0.75227 (18)	0.33978 (18)	0.45274 (12)	0.0229 (3)	
H6B	0.8057	0.3882	0.5101	0.027*	
H6A	0.7433	0.4145	0.4008	0.027*	
C7	0.84300 (17)	0.2068 (2)	0.42600 (11)	0.0233 (3)	
H7B	0.9501	0.2336	0.4360	0.028*	
H7A	0.8286	0.1207	0.4678	0.028*	

### Atomic displacement parameters (Å^2^)

**Table:**

	*U*^11^	*U*^22^	*U*^33^	*U*^12^	*U*^13^	*U*^23^
Zn1	0.01851 (12)	0.01557 (12)	0.01536 (12)	0.00055 (7)	0.00246 (8)	−0.00065 (7)
Cl1	0.02428 (19)	0.01691 (18)	0.0344 (2)	−0.00008 (14)	0.00302 (15)	−0.00614 (15)
Cl2	0.0279 (2)	0.02177 (19)	0.02037 (19)	−0.00225 (14)	−0.00132 (15)	0.00530 (13)
N1	0.0190 (6)	0.0199 (7)	0.0151 (6)	−0.0028 (5)	0.0018 (5)	−0.0018 (5)
N2	0.0185 (6)	0.0220 (6)	0.0164 (6)	−0.0032 (5)	0.0024 (5)	−0.0029 (5)
N3	0.0200 (6)	0.0183 (7)	0.0213 (7)	0.0008 (5)	0.0043 (5)	0.0008 (5)
C1	0.0209 (8)	0.0301 (9)	0.0247 (8)	−0.0056 (7)	0.0044 (6)	−0.0018 (7)
C2	0.0202 (7)	0.0201 (7)	0.0190 (7)	0.0001 (6)	0.0030 (6)	0.0031 (6)
C3	0.0240 (8)	0.0268 (8)	0.0181 (7)	−0.0012 (6)	0.0066 (6)	0.0011 (6)
C4	0.0247 (8)	0.0220 (7)	0.0173 (7)	0.0012 (6)	0.0036 (6)	0.0004 (6)
C5	0.0296 (8)	0.0311 (9)	0.0182 (7)	−0.0024 (7)	0.0036 (6)	−0.0043 (7)
C6	0.0198 (8)	0.0282 (9)	0.0211 (8)	−0.0062 (6)	0.0041 (6)	−0.0037 (6)
C7	0.0186 (7)	0.0313 (9)	0.0197 (7)	0.0020 (6)	0.0011 (6)	0.0022 (7)

### Geometric parameters (Å, °)

**Table:**

Zn1—N1	2.0214 (13)	C1—H1B	0.9800
Zn1—N3	2.0461 (14)	C2—C3	1.390 (2)
Zn1—Cl1	2.2266 (6)	C3—C4	1.382 (2)
Zn1—Cl2	2.2512 (6)	C3—H3	0.9500
N1—C2	1.340 (2)	C4—C5	1.492 (2)
N1—N2	1.3679 (18)	C5—H5B	0.9800
N2—C4	1.348 (2)	C5—H5A	0.9800
N2—C6	1.463 (2)	C5—H5C	0.9800
N3—C7	1.489 (2)	C6—C7	1.519 (2)
N3—H3A	0.9200	C6—H6B	0.9900
N3—H3B	0.9200	C6—H6A	0.9900
C1—C2	1.496 (2)	C7—H7B	0.9900
C1—H1A	0.9800	C7—H7A	0.9900
C1—H1C	0.9800		
			
N1—Zn1—N3	96.88 (6)	C3—C2—C1	128.97 (14)
N1—Zn1—Cl1	113.62 (4)	C4—C3—C2	106.55 (14)
N3—Zn1—Cl1	114.24 (4)	C4—C3—H3	126.7
N1—Zn1—Cl2	114.15 (4)	C2—C3—H3	126.7
N3—Zn1—Cl2	106.77 (4)	N2—C4—C3	106.56 (14)
Cl1—Zn1—Cl2	110.44 (3)	N2—C4—C5	122.65 (14)
C2—N1—N2	105.95 (12)	C3—C4—C5	130.79 (15)
C2—N1—Zn1	132.88 (11)	C4—C5—H5B	109.5
N2—N1—Zn1	120.99 (10)	C4—C5—H5A	109.5
C4—N2—N1	111.10 (13)	H5B—C5—H5A	109.5
C4—N2—C6	128.32 (13)	C4—C5—H5C	109.5
N1—N2—C6	120.56 (12)	H5B—C5—H5C	109.5
C7—N3—Zn1	115.47 (10)	H5A—C5—H5C	109.5
C7—N3—H3A	108.4	N2—C6—C7	112.70 (13)
Zn1—N3—H3A	108.4	N2—C6—H6B	109.1
C7—N3—H3B	108.4	C7—C6—H6B	109.1
Zn1—N3—H3B	108.4	N2—C6—H6A	109.1
H3A—N3—H3B	107.5	C7—C6—H6A	109.1
C2—C1—H1A	109.5	H6B—C6—H6A	107.8
C2—C1—H1C	109.5	N3—C7—C6	111.80 (13)
H1A—C1—H1C	109.5	N3—C7—H7B	109.3
C2—C1—H1B	109.5	C6—C7—H7B	109.3
H1A—C1—H1B	109.5	N3—C7—H7A	109.3
H1C—C1—H1B	109.5	C6—C7—H7A	109.3
N1—C2—C3	109.84 (14)	H7B—C7—H7A	107.9
N1—C2—C1	121.18 (14)		
			
N3—Zn1—N1—C2	−151.81 (14)	N2—N1—C2—C1	179.17 (14)
Cl1—Zn1—N1—C2	−31.54 (15)	Zn1—N1—C2—C1	−5.9 (2)
Cl2—Zn1—N1—C2	96.31 (14)	N1—C2—C3—C4	−0.41 (18)
N3—Zn1—N1—N2	22.52 (12)	C1—C2—C3—C4	−179.06 (16)
Cl1—Zn1—N1—N2	142.79 (10)	N1—N2—C4—C3	−0.02 (18)
Cl2—Zn1—N1—N2	−89.35 (11)	C6—N2—C4—C3	−178.15 (15)
C2—N1—N2—C4	−0.23 (17)	N1—N2—C4—C5	179.25 (14)
Zn1—N1—N2—C4	−175.91 (10)	C6—N2—C4—C5	1.1 (3)
C2—N1—N2—C6	178.07 (13)	C2—C3—C4—N2	0.25 (18)
Zn1—N1—N2—C6	2.38 (18)	C2—C3—C4—C5	−178.94 (17)
N1—Zn1—N3—C7	−0.89 (11)	C4—N2—C6—C7	122.45 (17)
Cl1—Zn1—N3—C7	−120.69 (10)	N1—N2—C6—C7	−55.52 (19)
Cl2—Zn1—N3—C7	116.93 (10)	Zn1—N3—C7—C6	−42.60 (16)
N2—N1—C2—C3	0.39 (17)	N2—C6—C7—N3	78.30 (17)
Zn1—N1—C2—C3	175.34 (11)		

### Hydrogen-bond geometry (Å, °)

**Table:**

*D*—H···*A*	*D*—H	H···*A*	*D*···*A*	*D*—H···*A*
N3—H3A···Cl2^i^	0.92	2.41	3.3073 (15)	165.
N3—H3B···Cl1^ii^	0.92	2.43	3.2620 (15)	150.

Symmetry codes: (i) −*x*+3/2, *y*−1/2, −*z*+1/2; (ii) −*x*+3/2, *y*+1/2, −*z*+1/2.
